# The Impact of Exercise and Virtual Reality Executive Function Training on Cognition Among Heavy Drinking Veterans With Traumatic Brain Injury: A Pilot Feasibility Study

**DOI:** 10.3389/fnbeh.2022.802711

**Published:** 2022-03-22

**Authors:** David L. Pennington, Jill V. Reavis, Monique T. Cano, Erica Walker, Steven L. Batki

**Affiliations:** ^1^Department of Psychiatry and Behavioral Sciences, University of California, San Francisco, San Francisco, CA, United States; ^2^San Francisco Veterans Affairs Health Care System (SFVAHCS), San Francisco, CA, United States; ^3^Northern California Institute for Research and Education (NCIRE), San Francisco, CA, United States; ^4^Department of Psychology, Palo Alto University, Palo Alto, CA, United States

**Keywords:** virtual reality, executive function, cognition, traumatic brain injury, alcohol use disorder, cognitive training, exercise, U.S. veterans

## Abstract

Executive function (EF) underlies self-control deficits in alcohol use disorder (AUD) and traumatic brain injury (TBI). Cognitive training is a promising adjunctive treatment targeting TBI- and AUD- related cognitive dysfunction. However, major limitations related to compliance and generalizability in the field of cognitive training exist. Physical activity is associated with enhanced cognitive performance across several executive functions and may enhance the benefits of cognitive training. Virtual reality provides multisensory embodied experiences which are likely to engage brain networks more efficiently than standard cognitive training systems, ultimately resulting in greater near- and far-transfer effects. This pilot study aimed to obtain feasibility data and a preliminary assessment of an enriched virtual reality (VR) EF training (EFT) intervention combined with exercise (NCT03786276). Using an 8-week randomized adaptive design study, 30 AUD treatment seeking U.S. Veterans completed nine sessions of exercise-only (*n* = 15) or gameplay control (*n* = 15) over 3 weeks, followed by a week-4 repeat assessment in Phase 1. Twenty-three participants completed and moved onto Phase II, where they completed up to nine sessions of VR-EFT plus exercise and completed a week-8 end-of-study assessment. Primary outcomes included feasibility to retain participants, usability, and satisfaction of using VR-EFT. Secondary and exploratory outcomes included within group assessment of change in cognitive function, alcohol use, alcohol craving, and post-concussive symptoms among the three treatment conditions.VR-EFT was feasible with moderate usability and high acceptability ratings.The most common VR-related adverse effect was motion sickness (*n* = 2/16, 12.5%). The VR-EFT condition was associated with significant improvement in inhibition-switching and visual scanning (both *p* < 0.05) during Phase II. Exercise-only was associated with significant improvements in cognitive inhibition, cognitive flexibility, reductions in alcohol craving, and number of standard alcohol drinks per week (all *p* ≤ 0.05). The gaming-control condition was associated with improvement in cognitive flexibility and visuospatial immediate recall (both *p* < 0.05) during Phase 1. Recruitment and retention of U.S. veterans with AUD and TBI into an exercise plus VR-EFT intervention is feasible, but technological barriers may impact usability. VR-EFT was associated with improvement in executive function domains that were targeted in as little as 3-week and nine sessions of VR-EFT exposure. Results are promising and indicate the need for a larger controlled investigation to assess the efficacy of VR-EFT to enhance treatment outcomes among AUD treatment-seeking U.S. veterans with co-occurring AUD and TBI.

**Clinical Trial Registration:**

www.ClinicalTrials.gov, Identifier: NCT03786276.

## Introduction

Executive function (EF) underlies successful self-regulation processes (Ilkowska and Engle, 2010; Hofmann et al., 2011) and difficulty with EF is commonly seen among individuals with alcohol use disorder (AUD), a population at disproportionate risk for traumatic brain injury (TBI; Drubach et al., 1993; Corrigan, 1995; Kreutzer et al., 1996; Hibbard et al., 1998; Bogner et al., 2001). U.S. Veterans are at greater risk for TBI and AUD relative to civilian counterparts (Brady et al., 2009; National Center for Health Statistics, 2019), and hazardous drinking can exacerbate ongoing TBI symptoms and increase vulnerability to future head injuries (Schumm and Chard, 2012). Conversely, TBI-related cognitive impairments increase one’s likelihood of engaging in hazardous alcohol use (Corrigan and Cole, 2008; Polusny et al., 2011; Miller, 2018; Jorge et al., 2019). Increased bidirectional risk between AUD and TBI is likely related to disruption of prefrontal cortical functioning in the areas of impulse control, reward expectation, and emotion regulation (Bjork and Grant, 2009). Many other conditions, including post-traumatic stress disorder (PTSD; Hoge et al., 2004; Corrigan and Cole, 2008; Brady et al., 2009; Gros et al., 2016), anxiety (Hoge et al., 2004), and depression (Hoge et al., 2004, 2008) also commonly co-occur among Veterans with TBI. Although effective interventions for the cognitive sequelae of TBI do exist, most are time-intensive for providers and patients (Cicerone et al., 2019) and very few studies have examined effective treatments specifically for Veterans with co-occurring AUD and TBI (Pennington et al., 2020). The lack of empirically supported treatments highlights the need to enhance interventions for these highly prevalent and costly co-occurring conditions and fill a gap in what’s known about addressing the overlapping cognitive sequalae of AUD and TBI in U.S. Veterans.

Cognitive training strategies that target cognitive dysfunctions have shown promise to restore cognitive alterations and help maintain abstinence in substance use disorders (Verdejo-Garcia et al., 2019) by enhancing neuroplasticity and supporting more adaptive decision-making ability (Houben et al., 2011; Verdejo-Garcia et al., 2019). Working-memory training (WMT) has been the most studied EF-based cognitive training tool (Verdejo-Garcia et al., 2019) and has demonstrated promise for near-transfer effects, including improved performance on related tasks of working-memory (Lechner et al., 2019) and some far-transfer effects related to reduced alcohol consumption among heavy drinkers following one month of training (Houben et al., 2011). WMT often involves a series of computerized tasks that require participants to repeatedly manipulate and recall stimuli (e.g., shapes, numbers) with increasing challenge. Beyond having limited evidence of far-transfer effects (e.g., robust change in alcohol use behavior), findings indicate that WMT is associated with design and compliance issues, as the tasks are often repetitive and require significant time to achieve the desired effects (Wanmaker et al., 2018). Thus, study retention is often poor, which raises concerns about WMT generalizability and its transferability toward other behaviors, including increased self-control over substance use (Wanmaker et al., 2018). Other 2-dimensional (2D) computer-based cognitive trainings have been shown to improve cognitive performance but failed to show transferability to real-world tasks (Ball et al., 2002) and there is little evidence to suggest that they offer an advantage compared to traditional paper methodologies, such as solving crossword puzzles or when compared to non-specific video games (Owen et al., 2010; Kable et al., 2017; Stanmore et al., 2017; Hampshire et al., 2019). These research gaps illuminate the need for more treatment studies on cognitive training overall, as well as the development of innovations to enhance cognitive training acceptability and potential long-term effectiveness.

Virtual reality (VR) technology has evolved substantially in the past several years, leading to the creation of low-cost, lightweight high-resolution headsets with sophisticated head tracking sensors to create a realistic sense of physical presence in virtual environments. Human cognition has been proposed to be deeply rooted in the body’s interactions with the world for the purpose of initiating and controlling physical action (Wilson, 2002; Raichlen and Alexander, 2017). While 2D screen-based systems fail to provide body-related sensory information (e.g., proprioception), VR environments provide multisensory embodied experiences which are likely to engage brain networks more efficiently than 2D systems, ultimately resulting in greater near- and far-transfer effects.

Emerging evidence supports the use of innovative interventions like VR to enhance cognitive functioning among individuals with AUD and cognitive impairment secondary to alcohol use (Gamito et al., 2021). Findings suggest that the utilization of VR technology can improve learning by helping focus attention through engagement in immersive, multisensory, realistic 3-D environments (Hoffman et al., 2004). Among U.S. veterans, it has been well established that simulated VR environments have been effective in promoting readiness and resilience (Parsons and Rizzo, 2008). Less is known, however, about the success of VR-based cognitive training interventions to enhance cognitive function and other outcomes among U.S. veterans with AUD and TBI (e.g., changes in substance use; Wanmaker et al., 2018; Lechner et al., 2019; Verdejo-Garcia et al., 2019).

Exercise has also been found to demonstrate considerable improvements in cognitive functioning (Hillman et al., 2008). Findings from behavioral (Kramer et al., 1999; Hillman et al., 2006) and neuroimaging (Colcombe et al., 2004) studies indicate that increased levels of physical activity are associated with enhanced cognitive performance across several executive functions, including attentional control, cognitive flexibility, and self-regulation. Exercise may result in even greater improvements in cognition if combined with cognitive training and when both are sufficiently demanding (Lauenroth et al., 2016). Evidence from multiple studies comparing combined cognitive training with physical activity to cognitive training alone supports the hypothesis that combined training produces greater overall benefits than the latter (Anderson-Hanley et al., 2012; Theill et al., 2013; Bamidis et al., 2014; Law et al., 2014; Zolyniak et al., 2014; Ballesteros et al., 2015; Joubert and Chainay, 2018). A study by Rahe et al. (2015) showed that while both cognitive training and combined cognitive and physical training showed significant gains in attention, only the combined training group showed a significant difference in divided attention and sustained improvement in a 1-year follow-up assessment. Physical activity combined with cognitive training may offer a greater potential benefit to cognitive functioning as both involve the recruitment of multiple resources and abilities rather than one alone.

Although not fully understood, physical activity may improve cognitive performance through a change in metabolic activity in the brain (cerebral blood flow resulting in increased oxygen and metabolism (Smith and Ainslie, 2017; Barnes and Corkery, 2018) or possibly through enhancing cerebral plasticity associated with increased brain-derived neurotrophic factor activation (Szuhany et al., 2015; Phillips, 2017; Walsh and Tschakovsky, 2018). Multiple reviews note the significant benefits of exercise in improving mood and decreasing anxiety (Giménez-Meseguer et al., 2020; Hu et al., 2020; Yoo, 2020; Ashdown-Franks et al., 2020; Smith and Merwin, 2021). In fact, physical activity has been helpful in reducing depression symptoms (Ashdown-Franks et al., 2020), alcohol consumption (including heavy drinking), and alcohol-related cravings among individuals with AUD (Roessler et al., 2017; Jensen et al., 2019; Hallgren et al., 2021). Pairing exercise with cognitive training may aid in improving alcohol treatment outcomes by impacting mechanisms related to mood, pleasure, and self-efficacy (Ashdown-Franks et al., 2020), in addition to neurological activation (Lauenroth et al., 2016).

Since improvement of cognitive function has the potential for enhancing AUD treatment effectiveness, we explored the potential benefit of a novel intervention that combines exercise with an enriched VR environment for delivering an executive function cognitive training intervention for heavy drinking U.S. Veterans with TBI. Our VR executive function training (VR-EFT) targets key aspects of EF—i.e., visual scanning, cognitive flexibility, cognitive inhibition, and processing speed—domains known to underlay significant EF deficits among individuals with AUD and TBI (Bates et al., 2013; Herrold et al., 2015; Verdejo-Garcia et al., 2019). The primary objectives of this study were to advance knowledge regarding the feasibility of delivering an exercise and VR-EFT intervention to understudied U.S. Veterans with co-occurring AUD and TBI, and to establish preliminary indication that VR-EFT is associated with EF improvement in this complex population. We also explore the extent to which exercise and VR-EFT generalize to other psychological and AUD treatment outcomes.

## Materials and Methods

### Participants

All participants provided written informed consent prior to the study and underwent procedures approved by the University of California, San Francisco, and the San Francisco Veterans Affairs Health Care System (SFVAHCS). All study procedures took place at SFVAHCS in San Francisco, CA. Participants were recruited from the SFVAHCS Addiction and Recovery Treatment Program *via* clinician referral or through research study fliers posted at the SFVAHCS between February 2019 and February 2020. The final sample included 30 Veterans with a documented history of TBI in the chronic stable phase of recovery (>6 months post-injury; American Congress of Rehabilitation Medicine (ACRM), Mild Traumatic Brain Injury Committee of the Head Injury Interdisciplinary Special Interest Group, 1993) and current (past year) moderate to severe AUD as defined by the DSM-5 (American Psychiatric Association, DSM-5 Task Force, 2013). In addition to meeting AUD diagnostic criteria, all Veterans reported “heavy” drinking based on NIH/NIAAA criteria (i.e., at least 15 standard drinks for men and at least eight drinks per week for women; Willenbring et al., 2009) for at least 1 week in the 90 days prior to consent. All participants were cleared for physical activity readiness by a study physician, expressed a desire to reduce or stop alcohol use and were receiving treatment for AUD at the SFVAHCS. Participants were free to access any standard psychosocial or pharmacologic treatments for AUD, TBI, or any other psychiatric or medical conditions.

The history of TBI was established using a structured clinical interview adapted from the Veterans Health Administration Comprehensive TBI Evaluation (previously the TBI Second Level Evaluation; Belanger et al., 2009; Pennington et al., 2020) to include a comprehensive assessment of lifetime history of head injury (in addition to most recent and most severe) and occurrence and duration of loss of consciousness, alteration of consciousness, and posttraumatic amnesia for each event. A history of TBI was considered present if a participant endorsed VA/Department of Defense and the ACRM (American Congress of Rehabilitation Medicine (ACRM), Mild Traumatic Brain Injury Committee of the Head Injury Interdisciplinary Special Interest Group, 1993) criteria of having a traumatically-induced physiological disruption of brain function as a result of an external force resulting in at least one of the following: a loss of consciousness, memory loss for events immediately before or after the event, alteration in mental state at the time of the event (e.g., disorientation, confusion, slowed thinking), or neurological deficit(s) (e.g., loss of balance/coordination, change in vision, weakness). Veterans were excluded if they were pregnant or attempting to conceive, had any unstable psychiatric (e.g., active psychotic symptoms) or medical issues (e.g., acute alcohol withdrawal) judged by study clinicians to pose unacceptable risks, had physical disability making it impossible to use a stationary recumbent bicycle (e.g., leg amputation), or concurrent participation in another AUD or TBI treatment studies. All assessments were administered by the Study PI (Clinical Psychologist) or by trained study staff under direct supervision of the Study PI. Random observations across time of cognitive assessments were conducted to ensure fidelity.

### Procedure

**Figure 1:**
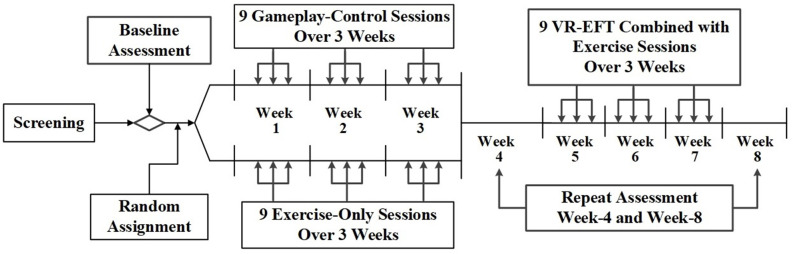
Virtual reality executive function training study design.

The primary goal of this study was to establish the feasibility of delivering an exercise and VR-EFT intervention to a difficult to recruit population of Veterans with TBI and AUD. Secondarily, we sought preliminary indication that VR-EFT is associated with improvement in targeted EF domains, and that this pattern of improvement would vary from within-group change observed in exercise-only or tablet-based gameplay conditions. Therefore, we chose to maximize the sample size within each of the three conditions and conducted an 8-week, randomized, adaptive design pilot study of a VR-based EFT combined with exercise compared to either exercise-only or tablet-based gameplay alone. Screening consisted of two to three visits during which participants completed the measures and interviews described below (Baseline Assessment). Veterans who met the study criteria were randomly assigned in a 1:1 ratio into a tablet-based gameplay condition or an exercise-only condition. Randomization was stratified on the total lifetime occurrence of TBIs (1 TBI vs. ≥ 2 TBIs) and balanced using computer-generated block randomization with permuted block sizes of six. Veterans were asked to complete nine 30-min sessions of the intervention they were assigned over the course of 3 weeks. These 3 weeks were followed by a 1-week washout period, which included repeat cognitive and other psychological assessments (week-4 assessment). Then, participants from both conditions were assigned to complete an exercise plus VR-EFT condition, which included up to nine 30-min VR-EFTsessions over a 3-week period. A final repeat post-treatment assessment occurred during the final week (i.e., week-8 assessment) after VR-EFT condition (Figure 1).

#### Materials

##### VR-EFT Exercise Condition

The VR-EFT exercise station consisted of a stationary recumbent bicycle, a set of gameplay controllers mounted on the recumbent bicycle handlebars, and an Oculus Rift VR headset. The VR-EFT task was developed by Blue Goji, a company that produces games designed to enhance neuroplasticity and promote user physical activity and engagement in VR gameplay. We utilized Blue Goji’s GoWings Safari, which engages and challenges cognitive domains of attentional scanning, processing speed, and cognitive inhibition and switching. Thus, it was considered appropriate to use as the VR-EFT intervention. This “game” is a path-based adventure game in which the user explores a wildlife environment. Users navigate through the virtual environment by pedaling a stationary bike. While doing so, they must scan and target animals and press a button on the handlebar-mounted controller to take the animal’s picture before they flee. If they see a “poacher,” they then alternate between the camera and net blaster to target and shoot the net blaster to capture the “poacher.” The faster the user pedals, the higher elevation they can gain in the environment enhancing field of view, and the faster they can move through the path. Certain animals are more difficult to spot, thus allotted higher scores. “Poachers” are associated with visual cues in the environment. Each environment includes several different collectible rewards, i.e., bananas (allows players to move faster in the environment without pedaling at a higher rate), camera film, and net blaster ammunition. Collecting items allows for the participant to increase the overall higher scores. Each session is randomly generated, spawning different animals and “poachers” in various locations. Participants can stay in the environment for an unlimited amount of time. The only limiting factors are film and ammunition, which can only be extended by finding collectibles. Participants’ overall score is a calculation of the photos and the number of poachers captured. There is feedback on achievement (score, distance traveled, photographs taken, and poachers captured) at the end of each session, tracked over multiple sessions. During the screening and prior to VR-EFT, each participant was given a tutorial demonstrating how to use the equipment in a non-specific VR environment. During the first training session, participants were oriented to the goals and gameplay. All sessions were conducted at the SFVAHCS under the supervision of a study staff member who was available to answer questions and provide guidance.

##### Gameplay Control Condition

To provide a non-VR gameplay control to our VR-EFT condition, participants could choose to play up to three non-specific 2D Blue Goji “racing” games, Speedbump, Super Moto X, or Row Your Boat, delivered on a mobile tablet. Each game required the user to manipulate a vehicle in 2-dimensional space down a “racetrack” using game controls presented on the touch screen tablet. The object of the gameplay generally included hitting targets with the user’s “vehicle” to increase speed and avoiding obstacles to win the race. These tasks targeted visual scanning and processing speed, but not higher-level components of executive function including cognitive inhibition or flexibility. During the screening, each participant was given a tutorial demonstrating how to play the games on the mobile tablet. During the first training session, participants were oriented to the goals and gameplay. Progress and advancement of gameplay were not tracked over time. All sessions were conducted at the SFVAHCS under the supervision of a study staff member who was available to answer questions and provide guidance.

##### Exercise-Only Condition

To provide an exercise condition that did not involve any cognitive challenge, participants used the identical stationary recumbent bicycle utilized for the VR-EFT condition, except without any VR-EFT equipment. The stationary recumbent bicycle tracked calories burned and distance traveled. During the first training session, participants were oriented to the use of the stationary recumbent bicycle. All sessions were conducted at the SFVAHCS under the supervision of a study staff member who was available to answer questions and provide guidance.

### Measures

#### Alcohol Use and Craving Measures

Alcohol use was assessed using the Time Line Follow Back (TLFB; Sobell et al., 1985; Sobell and Sobell, 1992) interview, which yields a total number of standard alcohol drinks per week. Alcohol craving-related obsessive thoughts and compulsions were measured using the Obsessive Compulsive Drinking Scale (OCDS; Anton et al., 1995) at baseline assessment, week-4 assessment, and week-8 end of study assessment. The OCDS is a 14-item self-report scale with a total score which can range from 0 to 56, an obsessive thoughts subscale that can range from 0 to 24, and a compulsive drinking subscale which can range from 0 to 32, with higher scores indicating a greater level of obsessive-compulsive drinking.

#### Alcohol and Substance Use Disorder Diagnosis and Depression/Anxiety Symptom Severity

All participants were administered the Substance Use Disorders sections of the Structured Clinical Interview for DSM-5 (American Psychiatric Association, DSM-5 Task Force, 2013) at baseline. The Alcohol Use Disorders Identification Test (AUDIT; Babor et al., 2001) was also administered to screen for harmful or hazardous alcohol use. The range of possible scores is from 0 to 40, where 0 indicates abstinence from alcohol, 1 to 7 indicates low-risk consumption, 8 to 14 suggests hazardous or harmful alcohol use, and scores of 15 or more indicate the likelihood of moderate to severe alcohol use disorder. Participants completed the Beck Depression Inventory (BDI-II; Beck et al., 1961), a 21-item inventory that ranges from 0 to 63 to characterize depression symptoms (0–13 minimal, 14–19 mild, 20–28 moderate, and 29–63 severe) and the Beck Anxiety Inventory (BAI; Beck et al., 1988) a 21 item inventory that ranges from 0 to 63 to characterize anxiety symptoms (0–7 mild, 8–15 mild, 16–25 moderate, 26–63 severe) at baseline.

#### Post-concussive Syndrome Symptoms

The Neurobehavioral Symptom Inventory (NSI; Department of Veterans Affairs, 2007), a 22-item measure designed to evaluate self-reported vestibular, somatic, cognitive, and affective post-concussive symptoms (e.g., headache, balance, nausea, etc.), was administered at baseline assessment, week-4 assessment, and week-8 end of study assessment. The NSI total score ranges from 0 to 88, with higher scores indicated a greater severity of post-concussive related symptoms. Subscale scores range from 0 to 12 for vestibular, 0 to 28 for somatic/sensory, 0 to 16 for cognitive, and 0 to 24 for affective symptoms. The Validity-10 scale was calculated to provide a measure of symptom validity assessment associated with the NSI, with acceptable scores being ≤22 (Vanderploeg et al., 2014).

#### Neurocognitive Assessment

The battery was developed to assess performance in cognitive domains commonly affected by heavy alcohol use and TBI. The battery contains only well-validated standardized instruments administered at baseline assessment, week-4 washout, and week-8 end of study assessment. Alternate forms were used for repeated administrations that are particularly susceptible to practice effects including the Hopkins Verbal Learning Test—Revised (HVLT-R; Brandt and Benedict, 2001) and brief visuospatial memory test—revised (BVMT-R; Benedict, 1997). Raw scores were converted to standardized scores or t-scores *via* appropriate normative data adjusted for age, ethnicity and/or education. Domains and constituent measures included: *Premorbid verbal intelligence*: Wechsler Test of Adult Reading (Venegas and Clark, 2011); *Working Memory*: WAIS-IV Arithmetic and Digit Span (Wechsler, 2008); *Visual Scanning*: D-KEFS Trail Making Test Condition 1 (Delis et al., 2004); *Cognitive Flexibility*: D-KEFS Trail Making Condition 4 (Letter-Number Sequencing) and Design Fluency Condition 3 (Switching Dots; Delis et al., 2004); *Cognitive Inhibition*: D-KEFS Color-Word Interference Test Condition 3 (Delis et al., 2004); *Cognitive Inhibition-Switching*: D-KEFS Color-Word Interference Test Condition 4 (Delis et al., 2004); *Processing Speed*: D-KEFS Trail Making Test, Condition 2 (Number Sequencing) and 3 (Letter Sequencing), D-KEFS Color-Word Interference Test Condition 1 (Color Naming), 2 (Word Naming), and DKEFS Design Fluency Condition 1 (Filled Dots) and 2 (Empty Dots; Delis et al., 2004); *Auditory-Verbal Recall*: Hopkins Verbal Learning Test-Revised (HVLT-R; Brandt and Benedict, 2001); and *Visuospatial Recall*: Brief Visuospatial Memory Test—Revised (BVMT-R; Benedict, 1997). The Reliable Digit Span (RDS) was calculated by summing the longest string of digits correctly recalled on forward plus backward trials (both trials correct) of WAIS-IV digit span to provide a measure of performance validity, with acceptable scores ≥7 (Schroeder et al., 2012).

#### Physical Activity and Readiness

All participants completed the physical activity readiness questionnaire for everyone (PAR-Q+), a 7-item screener followed by a two-page questionnaire that assesses for medical conditions that can limit physical readiness (Warburton et al., 2011). If a participant answered yes to one or more medical conditions that can limit physical activity, they were further assessed by a study physician using the physical activity readiness medical examination (PARmed-X). The study physician reviewed both the PAR-Q+ and PARmed-X prior to clearing aparticipant for unrestricted physical activity. Participants also completed the goden leisure-time exercise questionnaire (GLTEQ), a two-item questionnaire that measures the frequency of strenuous, moderate, and mild leisure physical activity performed for periods of 15 min or more over a usual week (Godin, 2011). This scale yields a leisure activity score that ranges from 0 to 13 (insufficiently active/edentary), 14 to 23 (moderately active), and 24 or more (active). Regardless of recent physical activity level, all participants were encouraged to start both the exercise-only and VR-EFT exercise conditions with low intensity and build up gradually to moderate intensity for the 30 min of participation and over the ninesessions of training.

#### Usability and Acceptability

The System Usability Scale (SUS; Brooke, 1996; Bangor et al., 2008), a validated measure of technology usability was administered at week-8 assessment to assess the usability of active VR-EFT. The client satisfaction questionnaire-8 (Larsen et al., 1979), designed to measure satisfaction with an clinical intervention, was used to assess satisfaction of our VR-EFT system at week-8 assessment.

#### End of Study Survey

Participants were asked to complete an author-created end of study survey. The survey included an item asking about the extent to which participants found the study’s interventions (e.g., VR plus exercise, iPad games, exercise only) helpful, with possible responses falling on a 5-point Likert scale ranging from “Not helpful at all” to “Very helpful”. Afterward, participants were asked in an open-text item to expand upon any aspects of the study that they disliked or found harmful. Participants were also asked in a checklist item to indicate whether they noticed any personal changes during the study (e.g., drank less, felt better mentally, other specified changes). At the end of the survey, we asked participants if they would recommend this study to other people (yes/no), followed by an open-text item to expand upon this response. Lastly, participants were encouraged to provide suggestions for future VR and exercise studies.

### Statistical Analysis

This pilot study was designed primarily to establish the feasibility of enrolling and retaining heavy drinking Veterans with TBI in an 8-week, randomized adaptive design trial of VR-EFT. To establish feasibility, we calculated study adherence and completion rate for each study condition. Results from the SUS and CSQ-8 were used to assess the usability and satisfaction of our VR-EFT system. Our secondary aim was to explore changes in performance in WM and other domains of cognitive function negatively affected by heavy alcohol use and TBI. All analyses are considered preliminary and hypothesis-generating. Therefore, the sample size was set for practical reasons and not driven by estimated effect sizes. However, power calculations based on a 0.05 alpha level reveal that 80% power (1-β) will detect a significant within group effect (VR-EFT condition) of change in cognitive function *via* F-testing of a medium to large effect size (0.15 ≤ Effect size f^2^ ≤ 0.35) and would require a total sample size of 7–33 (G-Power 3.1.9.2). In exploratory analyses, we examine if VR-EFT is associated with reduction in alcohol use (i.e., drinks per week), alcohol craving, and post-concussive symptoms.

All statistical analyses were performed with IBM SPSS Statistics Version 27 (IBM Corp, 2020). Baseline characteristics were compared using a one-way analysis of variance for continuous variables and the chi-square test (or Fisher’s exact test when cell sizes were small) for categorical variables. Continuously scaled repeated measures were analyzed with random-intercept linear mixed models using restricted maximum likelihood by SPSS’s MIXED procedure. An auto-regressive correlation structure was used in general linear models to adjust for repeated observations within participants. SPSS’s MIXED procedure allows for the inclusion of all available data. Therefore, no imputation methods were used to attempt to account for missing data among primary outcome variables in these modeling procedures.

Within group analyses included a fixed predictor of time; week-4 vs. week-8 for VR-EFT analyses, and baseline assessment vs. week-4 assessment for gameplay control and exercise-only conditions. In models assessing change in cognition, pre-morbid verbal intelligence (WTAR) was included as a covariate. Baseline week-0 cognitive performance was also included as a covariate in the respective model when examining VR-EFT related cognitive domain changes between week-4 and week-8 to account for potential changes in cognition during Phase I exercise-only and gameplay control conditions. Weekly drinks per week over the iteration of time associated with each condition (weeks 0–4 for exercise-only and gameplay control condition, or weeks 4–8 for VR-EFT condition) were examined as the primary variable under investigation for the alcohol use model. In the alcohol model, an aggregate of drinks per week over the 90 days prior to consent was modeled as a covariate. All analyses were intent-to-treat and used all possible observations for participants who were enrolled and completed at least one session of each condition. No imputation methods were used to attempt to account for missing data beyond endpoints in which subjects were no longer participating in the study (i.e., withdrawn and lost to follow-up). An alpha level of *p* ≤ 0.05 was considered significant.

## Results

### Patient Characteristics and Baseline Cognitive Function

Baseline characteristics are shown in Table 1. Of the 30 participants, 15 were randomly assigned to the exercise-only condition and 15 to the gameplay-control condition at study enrollment. Of those, 23 participants completed phase I and entered the VR-EFT condition (phase II). The participants allocated to the three treatment conditions did not differ on any baseline characteristics or baseline cognitive domain scores (Table 2). The baseline VR-EFT scores in Table 2 reflect the 16 participants that completed the VR-EFT condition. As a measure of performance and symptom validity, we calculated the reliable digit span (RDS) and Validity-10 scale, respectively (Schroeder et al., 2012; Vanderploeg et al., 2014). Both scales showed good validity (RDS ≥ 7, Validity-10 ≤ 22, Table 1). Although not a requirement for study inclusion, all participants indicated experience using a mobile tablet for gameplay, and no participants indicated previous use of virtual reality equipment.

**Table 1:** Participant characteristics (Means ± Standard Deviation).

	**Exercise-Only**	**Gameplay control**	**VR-EFT**
n (female)	13 (2)	12 (3)	18 (5)
Age [years]	49.2 ± 11.0	52.8 ± 7.7	52.3 ± 9.6
Education [years]	14.3 ± 1.9	13.1 ± 3.8	12.7 ± 3.6
Hispanic or Latino	5 (33%)	1 (7%)	4 (17%)
Race			
Caucasian	8 (53%)	8 (53%)	11 (48%)
African American	5 (33%)	6 (40%)	11 (48%)
Native American	1 (7%)	0 (0%)	1 (4%)
Mixed Race	1 (7%)	1 (7%)	0 (0%)
Combat Exposed, n(%)	4 (27%)	1 (7%)	1 (4%)
GLTEQ Leisure Activity Score*	26.9 ± 21.3	61.8 ± 38.3	42.6 ± 38.6
Comorbid Substance Use Disorder, n(%)	8 (53%)	9 (60%)	12 (52%)
BDI	15.4 ± 11.3	16.1 ± 10.36	15.1 ± 10.8
BAI	15.1 ± 10.4	14.2 ± 10.6	14.5 ± 9.7
AUDIT	16.4 ± 7.8	16.8 ± 8.2	16.1 ± 7.8
Drinks per Week^$^	30.8 ± 29.2	28.9 ± 30.9	24.7 ± 22.7
Heavy Drinking Days per Week^$^	2.9 ± 2.7	2.8 ± 2.7	2.7 ± 2.6
Obsessive Compulsive Drinking Scale	13.7 ± 6.9	13.4 ± 8.2	10.1 ± 6.8
Obsessive Subscale	6.1 ± 4.2	5.1 ± 3.8	4.4 ± 3.2
Compulsive Subscale	7.6 ± 3.2	8.3 ± 4.8	5.7 ± 3.9
Mild TBI	12 (80%)	11 (73%)	18 (78%)
Moderate TBI	0	2 (13%)	2 (9%)
Severe TBI	3 (20%)	2 (13%)	3 (13%)
TBI count total	69	54	78
Blunt Trauma	27	26	36
Blast	12	3	12
Fall	20	21	19
Motor Vehicle	10	15	11
Post-Injury Interval	13.7 ± 13.4	13.9 ± 16.7	15.1 ± 15.2
Neurobehavioral Symptom Inventory	19.4 ± 13.1	22.5 ± 12.3	16.2 ± 15.1
Vestibular	2.3 ± 1.9	2.4 ± 2.0	1.9 ± 2.5
Somatic	5.3 ± 4.5	5.5 ± 3.9	3.9 ± 4.4
Affective	6.9 ± 5.7	8.1 ± 4.5	6.2 ± 5.6
Cognitive	4.9 ± 3.9	6.4 ± 4.7	4.3 ± 4.1
RDS	9.4 ± 2.1	9.7 ± 2.4	9.4 ± 2.5
Validity-10	8.1 ± 5.7	9.1 ± 6.4	8.5 ± 5.6

Abbreviations: GLTEQ, Goden Leisure-Time Exercise Questionnaire; BDI, Beck Depression Inventory; BAI, Beck Anxiety Inventory; AUDIT, Alcohol Use Disorder Identification Test; TBI, traumatic brain injury; RDS, Reliable Digit Span; heavy drinking day (>4 standard alcoholic drinks for men, >3 standard alcoholic drinks for women). Drink consumption was averaged over 90 days preceding study consent. *p ≤ 0.05, ^$^standard alcoholic drink is defined as containing 13.6 g of pure alcohol. Post-Injury Interval: years between most recent TBI and baseline assessment.

**Table 2:** Secondary outcome: within group effects of cognitive function.

**Cognitive domain**		**Baseline Mean±SD**	**Week-4 Mean±SD**	**Week-8 Mean±SD**	***p*-Value**	**β**	**95% CI**
Working Memory (SS)	Exercise-Only^$^	9.2 ± 2.0	9.0 ± 2.5	-	0.219	−0.07	−0.77–0.63
	Gameplay Control^$^	9.5 ± 2.4	9.0 ± 2.9	-	0.402	0.75	−1.11–2.61
	VR-EFT^$^	9.2 ± 2.3	8.9 ± 2.6	9.0 ± 2.7	0.163	−0.58	−1.42–0.26
Visual Scanning (SS)	Exercise-Only	9.1 ± 3.2	9.3 ± 3.3	-	0.432	−0.52	−1.93–0.89
	Gameplay Control	11.4 ± 2.4	10.7 ± 2.7	-	0.219	0.97	−0.67–2.60
	VR-EFT^#^	10.4 ± 3.1	10.0 ± 3.0	11.1 ± 2.5	0.020	−1.54	−2.78 to −0.29
Cognitive Flexibility (SS)	Exercise-Only	9.4 ± 2.7	10.4 ± 1.7	-	0.027	−1.30	−2.42 to −0.18
	Gameplay Control	8.3 ± 2.9	10.1 ± 2.3	-	0.050	−1.55	−3.09 to −0.01
	VR-EFT^#^	8.8 ± 2.5	10.3 ± 2.0	9.8 ± 2.3	0.815	−0.06	−0.64–0.51
Cognitive Inhibition (SS)	Exercise-Only	8.1 ± 4.0	10.3 ± 3.6	-	0.004	−2.42	−3.91 to −0.95
	Gameplay Control	10.1 ± 4.1	11.6 ± 2.1	-	0.462	−0.55	−2.16–1.06
	VR-EFT^#^	9.7 ± 4.1	11.0 ± 3.0	10.5 ± 3.3	0.907	−0.04	−0.82–0.73
Cognitive Inhibition-Switching (SS)	Exercise-Only	8.7 ± 3.2	10.3 ± 2.2	-	0.093	−1.51	−3.31–0.29
	Gameplay Control^$^	10.3 ± 3.2	11.0 ± 1.9	-	0.693	0.20	−0.89–1.29
	VR-EFT^#^	10.2 ± 3.0	10.6 ± 2.0	11.0 ± 2.3	0.018	−0.90	−1.63 to −0.17
Processing Speed (SS)	Exercise-Only	9.1 ± 2.1	9.8 ± 1.8	-	0.051	−0.92	−1.85–0.01
	Gameplay Control	10.2 ± 2.8	10.6 ± 1.9	-	0.858	0.07	−0.73–0.86
	VR-EFT^#^	9.8 ± 2.2	10.2 ± 1.9	10.3 ± 2.1	0.533	−0.22	−0.96–0.52
Auditory-Verbal Immediate Recall	Exercise-Only	38.5 ± 11.0	41.4 ± 14.6	-	0.342	−2.86	−9.18–3.45
	Gameplay Control	36.4 ± 10.9	39.6 ± 16.1	-	0.618	−1.91	−10.08–6.25
	VR-EFT^#^	38.1 ± 11.5	40.5 ± 15.0	42.0 ± 12.2	0.909	−0.09	−4.65–5.19
Auditory-Verbal Delayed Recall	Exercise-Only	36.4 ± 13.3	36.7 ± 16.8	-	0.909	−0.38	−7.53–6.77
	Gameplay Control	35.1 ± 11.6	36.2 ± 16.6	-	0.970	−0.14	−8.32–8.03
	VR-EFT^#^	36.3 ± 12.6	36.5 ± 16.2	40.8 ± 14.2	0.542	−1.59	−7.01–3.83
Visuospatial Immediate Recall	Exercise-Only	37.1 ± 11.1	42.2 ± 12.5	-	0.166	−4.18	−10.36–2.01
	Gameplay Control	33.7 ± 11.7	43.3 ± 13.1	-	0.001	−8.24	−12.37 to −4.11
	VR-EFT^#^	36.2 ± 12.1	42.7 ± 12.5	42.2 ± 10.9	0.847	−0.62	−7.30–6.05
Visuospatial Delayed Recall	Exercise-Only	38.3 ± 11.1	44.9 ± 14.5	-	0.101	−4.80	−13.81–1.43
	Gameplay Control	36.9 ± 12.6	43.3 ± 11.6	-	0.063	−4.86	−10.04–0.32
	VR-EFT,^#^	38.5 ± 11.3	44.1 ± 12.8	41.5 ± 14.2	0.696	1.03	−4.50–6.55

*Abbreviations: SS, Scaled score; β, beta; CI, confidence interval; significant covariate predictor of ^$^ Wechsler Test of Adult Reading, ^#^baseline cognitive function; Auditory-Verbal and Visuospatial Recall scores are reported at standard t-scores; Baseline assessment scores for VR-EFT represent means ± standard deviations of the 23 participants who were allocated to Phase II*.

### Study Completion and Adherence

**Figure 2:**
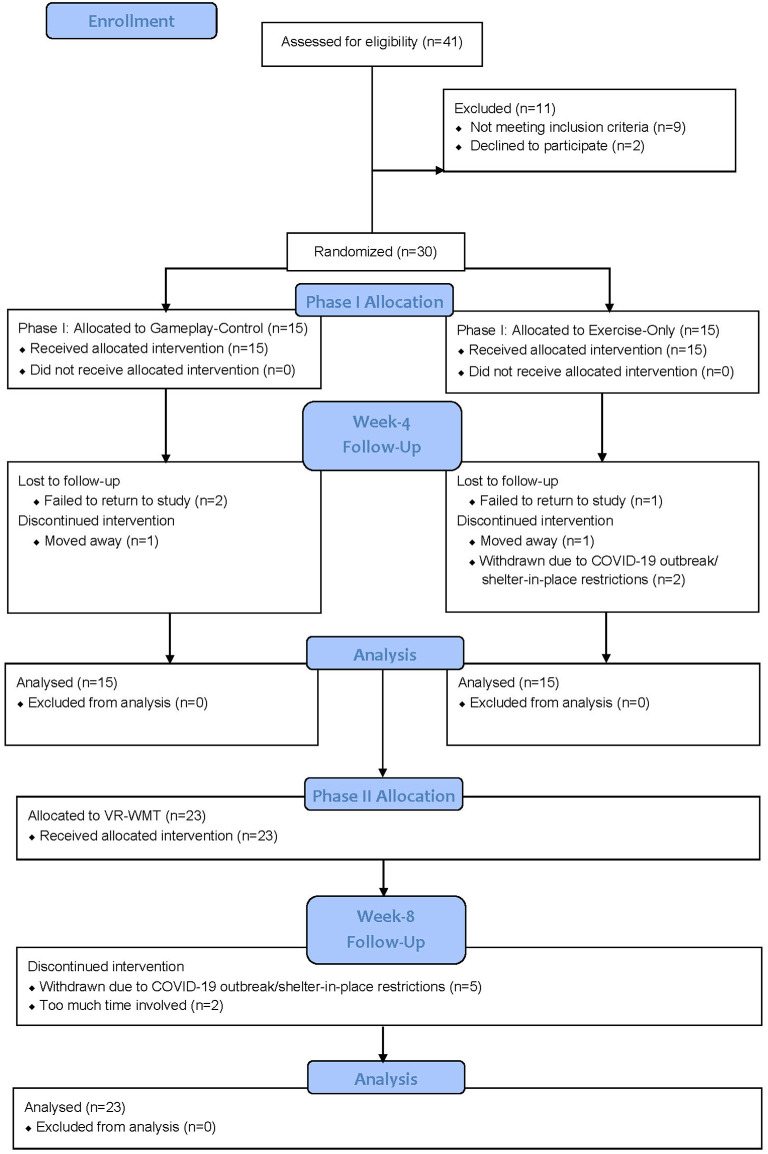
Consort flow diagram.

Subject flow is illustrated in Figure 2. Of the 30 participants, 28 had ≥2 lifetime TBIs. The two participants with only 1 TBI were randomized to the gameplay control condition in Phase I. Study completion for Phase I was defined as being present for the week-4 assessment visit and completion for Phase II was defined as being present for the week-8 post-treatment assessment visit. During Phase I, 11/15 (73%) completed the exercise-only bike condition, 1/15 (7%) was lost to follow-up, 1/15 (7%) moved away, and 2/15 (13%) were withdrawn due to COVID-19 outbreak/shelter-in-place restrictions. Also, in Phase I, 12/15 (80%) completed the gameplay-control condition, 2/15 (13%) were lost to follow-up and 1/15 (7%) moved away. In total, 23/30 (77%) participants completed Phase I and began Phase II. During Phase II, 16/23 (70%) completed the VR-EFT condition of the study, 2/23 (9%) dropped out due to “too much time involved” and 5/23 (22%) were withdrawn due to COVID-19 outbreak/shelter-in-place restrictions. No participants dropped out due to reported adverse effects related to study conditions. Of the 30 randomized participants, 16/30 (53%) completed the full study.

The mean number of sessions completed did not significantly differ between treatment conditions (*p* = 0.112). Participants completed 7.1 ± 2.1 exercise only sessions, 7.4 ± 2.4 gameplay control sessions, and 5.7 ± 2.8 VR-EFT sessions in each condition. Participants were asked to use the VR-EFT station, stationary bike, or mobile tablet for gameplay for at least 30 minutes per session but were allowed to discontinue or extend use as desired. Participants utilized the VR-EFT station (23.1 ± 7.1 min) for significantly less time (*p* = 0.02) than the mobile tablet for gameplay (27.5 ± 2.7 min). Duration of use per exercise bike session (26.0 ± 4.5 min) did not differ from VR-EFT or gameplay only conditions. Average calories burned per session were significantly (*p* < 0.01) higher for the exercise-only condition (97.8 ± 34.0 calories) vs. VR-EFT condition (65.0 ± 25.3 calories).

### Usability and Acceptability

The VR-EFT system received a SUS rating of 70% ± 20% (45% minimum and 100% maximum). An acceptable standard is 70%, rating the system at least “moderate” (Brooke, 1996; Bangor et al., 2008). Scores of less than 50% should be cause for significant concern and are judged to be unacceptable, whereas scores 70%–90% are promising, and scores above 90% are viewed as superior (Bangor et al., 2008). Of the 16 Phase II study completers, 2/16 (12.5%) had a SUS rating below 50%, 6/16 (37.5%) rated the system between 50% and 69%, 4/16 (25%) rated the system between 70% and 90%, and 4/16 (25%) rated the system above 90%.

Client satisfaction (CSQ-8) with the VR-EFT system was rated 30.7 ± 1.8 (26 minimum and 32 maximum). A standard rating of 24 is generally considered acceptable (Larsen et al., 1979). Zero items on the CSQ-8 were rated with a score of 1 (e.g., poor) by any participant. The highest-rated was item 4, “If a friend were in need of similar help, would you recommend this intervention to them?” All participants responded, “Yes, Definitely.” No items had a mean score of less than 3.6 ± 0.63 out of a possible total item score of 4. The lowest scored item was item 6, which states “Have the services you received helped you to deal more effectively with your problems?” The average response on item 6 was between “Yes, they helped, ” to “Yes, they helped a great deal.”

### End of Study Questionnaire

Responses on the end of study questionnaire indicated overall favorable impressions of VR-EFT (3.64 ± 1.45 on a 5-point scale) and gameplay control condition (3.14 ± 1.56), with the highest ratings found for exercise-only (4.07 ± 1.03). Exercise-only was rated significantly higher than gameplay control condition (*p* = 0.05). No other significant differences were observed. When asked about any dislikes/harmful aspects of the study, two participants reported VR-related motion sickness and one reported intermittent technical issues (e.g., “the VR was not always working properly”). Among those responding to the item about any personal changes during the study (*n* = 15), 20% (*n* = 3) reported no changes, 20% (*n* = 3) reported that they quit drinking, 26.67% (*n* = 4) reported that their appearance improved, 33.33% (*n* = 5) reported that they drank less, 60% (*n* = 9) reported that they felt better mentally, and 66.67% (*n* = 10) reported that they felt better physically. All respondents reported that they would recommend the study to other people, with one participant stating they believe it could help reduce drinking and others commenting on it being “interesting,” “fun,” “challenging,” and a “possible learning experience.” When asked about suggestions for future VR and exercise studies, respondents suggested moving the exercise bike to a quieter location, slowing down the speed of the game, more advanced equipment (e.g., VR controllers, exercise bike), and running the study longer than the 8-week period.

### Secondary Analyses: Within Group Change in Cognitive Function

Participants in the VR-EFT condition performed significantly better on inhibition switching (*F*_(1,14)_ = 7.18, *p* = 0.018, beta [β] = −0.90; 95% CI = −1.63 to −0.18) and visual scanning (*F*_(1,13)_ = 8.05, *p* = 0.014, beta [β] = −1.58; 95% CI = −2.79 to −0.37) at week-8 post assessment compared to week-4 assessment (Table 2).

Participants in the exercise-only condition performed significantly better on cognitive inhibition (*F*_(1,11)_ = 13.02, *p* = 0.004, beta [β] = −2.43; 95% CI = −3.91 to −0.95), cognitive flexibility (*F*_(1,10)_ = 6.69, *p* = 0.027, beta [β] = −1.30; 95% CI = −2.42 to −0.18), and marginally better on processing speed (*F*_(1, 10)_ = 4.89, *p* = 0.051, beta [β] = −0.92; 95% CI = −1.85–0.01) at week-4 assessment compared to baseline assessment (Table 2).

Participants in the gaming-control condition performed significantly better on visuospatial immediate recall (*F*_(1,10)_ = 19.54, *p* = 0.001, beta [β] = −8.24; 95% CI = −12.36 to −4.11) and cognitive flexibility (*F*_(1,11)_ = 4.82, *p* = 0.050, beta [β] = −1.55; 95% CI = −3.10 to −0.01) at week-4 assessment compared to baseline assessment (Table 2).

### Exploratory Analyses: Within Group Change in Alcohol Use, Craving and Post-concussive Symptoms

During the exercise-only condition, participants significantly reduced number of drinks per week during weeks 1–4 of the study (*F*_(1,43)_ = 8.30, *p* = 0.006, beta [β] = −4.30; 95% CI = −7.30 to −1.30) and reported significantly less alcohol craving (OCDS Total: *F*_(1,10)_ = 26.37, *p* < 0.001, beta [β] = 5.32; 95% CI = 3.03–7.62; Obsessive Subscale: *F*_(1,10)_ = 12.20, *p* = 0.006, beta [β] = 2.40; 95% CI = 0.88–3.93; Compulsive Subscale: *F*_(1,10)_ = 19.85, *p* = 0.001, beta [β] = 2.84; 95% CI = 1.43–4.25) (Table 3). Participants in the gameplay control condition also reported less alcohol craving, but only on the compulsive subscale of the OCDS (*F*_(1,10)_ = 10.20, *p* = 0.010, beta [β] = 1.57; 95% CI = 0.48–2.67) (Table 3). There were no significant changes in alcohol use or alcohol craving associated with the VR-EFT condition. There were no significant changes in post-concussive symptoms associated with any of the three conditions.

**Table 3:** Exploratory outcome: within group effects of alcohol use, craving, and postconcussive symptoms.

**Psych domain**		**Baseline Mean±SD**	**Week-4 Mean±SD**	**Week-8 Mean±SD**	**p-Value**	**β**	**95% CI**
Drinks Per Week	Exercise-Only^$^	28.0 ± 26.7	9.1 ± 15.2	-	0.006	−4.30	−7.30 to −1.29
	Gameplay Control^$^	20.4 ± 27.5	16.9 ± 18.4	-	0.533	−0.76	−3.42–1.89
	VR-EFT	24.6 ± 26.1	13.6 ± 17.1	9.6 ± 14.2	0.308	−0.79	−2.35–0.78
OCDS Total	Exercise-Only	13.7 ± 6.9	9.5 ± 4.2	-	0.000	5.32	3.03–7.62
	Gameplay Control	13.4 ± 8.2	10.8 ± 8.9	-	0.099	1.88	−0.42–4.17
	VR-EFT	13.8 ± 7.0	10.1 ± 6.8	9.2 ± 7.4	0.729	0.39	−1.96–2.74
OCDS Obsessive Subscale	Exercise-Only	6.1 ± 4.2	4.3 ± 2.8	−	0.006	2.40	0.88–3.93
	Gameplay Control	5.1 ± 3.8	4.6 ± 3.6	-	0.642	0.31	−1.14–1.76
	VR-EFT	5.7 ± 3.8	4.4 ± 3.2	3.8 ± 3.6	0.700	0.22	−0.98–1.42
OCDS Compulsive Subscale	Exercise-Only	7.6 ± 3.2	5.2 ± 2.2	-	0.001	2.84	1.43–4.25
	Gameplay Control	8.3 ± 4.8	6.3 ± 5.3	-	0.010	1.57	0.48–2.67
	VR-EFT	8.1 ± 3.7	5.7 ± 4.0	5.3 ± 4.6	0.799	0.20	−1.45–1.85
NSI Total	Exercise-Only	19.4 ± 13.1	15.3 ± 16.0	-	0.170	5.41	−2.70–13.52
	Gameplay Control	22.5 ± 12.3	17.1 ± 15.0	-	0.283	3.51	−3.36–10.38
	VR-EFT	20.3 ± 11.7	16.2 ± 15.1	13.2 ± 10.5	0.668	0.90	−3.53–5.33

*Abbreviations: β, beta; CI, confidence interval; OCDS, Obsessive Compulsive Drinking Scale; NSI, Neurobehavioral Symptom Inventory; significant covariate predictor of ^$^past 90-day drinks per week; Baseline scores for VR-EFT represent means ± standard deviations of the 23 participants who were allocated to Phase II*.

## Discussion

This is the first study to examine the effects of a virtual reality cognitive training and exercise intervention on executive function, alcohol use, and post-concussive symptoms in Veterans with traumatic brain injury and alcohol use disorder. Delivering an exercise and VR-EFT intervention to U.S. Veterans with co-occurring AUD and TBI was feasible with moderate usability and high acceptability ratings. We observed a preliminary indication that VR-EFT is associated with cognitive improvement in domains related to the specific cognitive challenges of our VR-EFT condition, i.e., visual scanning and cognitive inhibition-switching. In addition, non-specific gameplay was associated with improvements in cognitive flexibility and visuospatial recall. We also observed significant improvements in cognitive flexibility and reductions in the total number of drinks per week and alcohol craving in our exercise-only condition. Although these changes may have been partially driven by natural recovery, practice effects, and nonspecific effects, such as participant expectancy, interaction with study staff, and close monitoring, these findings lend support for further investigation into the beneficial effects of VR-based cognitive training and exercise for cognitive improvement and alcohol use reduction in this understudied population of U.S. Veterans with AUD and TBI.

Heavy alcohol use can both predispose individuals to TBI and exacerbate existing TBI symptoms, thereby increasing vulnerability to future injuries (Schumm and Chard, 2012). Conversely, the cognitive impairments that accompany TBI can also increase predisposition to heavy alcohol use (Corrigan and Cole, 2008; Polusny et al., 2011; Jorge et al., 2019). The 30 Veteran participants in this study reported 123, mostly mild TBI’s (*n* = 25, 75%), a notably high TBI count, especially for a sample with minimal combat exposure (*n* = 5/30, 17%). Only 2/30 (7%) reported a single TBI. A comprehensive TBI evaluation includes reviewing a history of TBI occurrence over the lifetime. Many of the injuries reported in the current sample are from falls, blunt traumas, and motor vehicle accidents that occurred either pre- or post-military service. In the general population, 30%–50% of patients treated for TBI report intoxication at the time of injury with binge drinking being a major risk factor for falls and accidents that are associated with brain trauma (for review see Weil et al., 2018). In general, the rate of TBIs among those with substance use disorders is high, although the true incidence is unknown because there is no standard practice for evaluating TBI in substance use treatment settings (Miller, 2018). The high rates of TBI in this study sample highlight the importance of assessing for TBI in AUD treatment settings and developing interventions such as VR-EFT to address the unique and overlapping symptoms associated with TBI and AUD in this vulnerable population.

VR-EFT, which incorporated physical exercise, was generally well-tolerated and feasible to use. Although the VR-EFT condition involved the use of additional hardware and required additional steps to set up and use than the other two conditions (gameplay- and exercise-only), the total number of sessions that participants completed did not significantly differ between conditions. However, participants did spend about 4.5 minutes more using games delivered on the mobile tablet than they did utilizing the VR-EFT station, even though VR-EFT was indicated as being more helpful than tablet-delivered games. Participants did not spend significantly more time using the exercise bike than they did using VR-EFT or playing games on the mobile tablet, even though the exercise-only condition was rated as the most helpful of the three interventions. Participants did burn more calories during the exercise-only condition compared to exercise plus VR-EFT. The additional complexity of completing cognitive training in VR while on an exercise bike likely contributed to decreased bike intensity. This was contrary to the intended design, which may have contributed to diminished cognitive improvement during the VR-EFT condition.

VR-EFT was associated with motion sickness in 12.5% of participants (*n* = 2/16), an adverse event that is not uncommon during VR use (Kolasinski, 2014). Blue Goji’s GoWings Safari game is designed with vertical movement by the user’s game character. As bicycle speed increases and reduces, the user’s gaming character rises and falls in 3-D space from the perspective of the user. Motion sickness in VR is thought to be related to sensory conflicts when a user’s perception of self-motion is incongruent with sensory inputs from the vestibular system (Reason and Brand, 1975; Johnson, 2005; Kolasinski, 2014). Forward movement in Blue Goji’s GoWings Safari is associated with the speed of stationary bike pedaling as well as rising and falling, the latter of which is not congruent with typical biking experience or vestibular input from the user’s perspective. This may have resulted in complaints of motion sickness. Future VR-based cognitive training tasks may want to pay special consideration to motions/movements that can be disorienting to the user to improve user satisfaction and desire to engage with the training task.

Twenty percent or more of respondents that completed the VR-EFT reported that they quit drinking or drank less, their appearance improved, and that they felt better mentally and physically. All respondents noted that they would recommend exercise plus VR-EFT to other people, stating that it was “interesting,” “fun,” and “challenging.” In fact, the system received a rating of 70% on the system usability scale which is considered “promising” by industry standards, and a score of 30.7 on the client satisfaction questionnaire, which is close to the highest possible score of 32 (Larsen et al., 1979; Brooke, 1996; Bangor et al., 2008). Intermittent technical issues, distractions in the physical environment, speed of the game, and issues with equipment (e.g., VR controllers not always working and exercise bikes not easily syncing with the game) all contributed to negative aspects of the participants’ experience. Improving user experience and reducing the occurrence of technical issues may likely enhance engagement with VR-based cognitive training interventions.

The VR-EFT condition was associated with near-transfer effects to two cognitive domains that our training targeted, visual scanning and cognitive inhibition-switching. Neither of these cognitive domains was associated with improvement during Phase I of the study that exposed participants to exercise-only or the gameplay control condition. The primary tasks in GoWings Safari are to scan the visual environment, inhibit and switch, or activate a tool (e.g., camera or net blaster) to capture a target (e.g., animal or “poacher”). Improvements in both visual scanning and inhibition-switching during Phase II and not Phase I may reflect the specific training received during our VR-EFT condition and are less likely related to practice or other non-specific effects of the study. Particularly as the measures used in the study are well-validated standardized instruments that utilize alternative forms when necessary. Still, given the lack of direct group comparison, the cognitive gains observed during Phase II may be due to other non-specific effects and cannot be ruled out.

Visual scanning was measured using a visual attention task (Delis et al., 2007) and studies consistently demonstrate that many people with SUD exhibit cognitive deficits in attention (Verdejo-Garcia et al., 2019). Long-term alcohol use is associated with deficits in visual scanning (Ciesielski et al., 1995), and accuracy during attention is a consistent predictor of treatment retention (Dominguez-Salas et al., 2016) and relapse (Rolland et al., 2019) in SUD studies. Inhibition-switching is also consistently associated with deficits in SUD (Verdejo-Garcia et al., 2019) and these deficits are associated with lower quality of life improvement during early treatment (Rubenis et al., 2018). There have been a small number of studies that have focused on inhibition control training (Verdejo-Garcia et al., 2019) that have shown modest reductions in alcohol use, but none have been conducted among treatment seekers (Verdejo-Garcia et al., 2019). Our study represents the first VR-EFT study that targets inhibition-switching among a sample of treatment-seeking U.S. Veterans.

Similarly, cognitive bias modification (CBM) studies have targeted attentional bias training for substance-related cues. One attentional bias alcohol study has shown a delay in time to relapse (but not relapse rates) relative to controls who received sham training (Schoenmakers et al., 2010) and a second study showed reduced alcohol relapse rates at a one-year follow-up relative to a sham training condition in a sample of AUD treatment seekers (Rinck et al., 2018). These studies were attempting to train attentional bias specific to alcohol-related cues and were different from the current study in that the target of VR-EFT was a general attentional skill not associated with specific alcohol-related imagery. Studies of attentional bias modification have thus far shown evidence for reduced AUD symptoms, but not evidence for reductions in attentional bias (Heitmann et al., 2018). Our study provides preliminary evidence showing that attentional scanning can be targeted with a VR-based general attentional training approach vs. an attentional bias targeting a specific substance. However, our small pilot study did not show far transfer effects, i.e., an indication for reduction in alcohol use or alcohol craving during and immediately following VR-EFT.

During Phase I, we also saw significant improvement in cognitive inhibition associated with the exercise-only condition, but not gameplay control. A recent meta-analysis reported small to moderate effects of exercise on executive function, including inhibitory control in healthy populations (Ludyga et al., 2016). We also saw a significant reduction in alcohol use and alcohol craving associated with exercise-only condition in our study. Among people with methamphetamine use disorder exercise has been shown to improve inhibitory control and reduce craving (Wang et al., 2015), but findings for the benefits of exercise for AUD have only consistently demonstrated improved mood and physical health outcomes, but not reduced alcohol use, alcohol craving, or improved cognitive function (Zschucke et al., 2012; Hallgren et al., 2017). This lack of effect may be due to the limited number of longitudinal, well-controlled studies on the impact of exercise on AUD symptom outcomes, including cognition (Hallgren et al., 2017). As such, there is still enthusiasm for continued work in understanding the benefits and potential mechanisms underlying neurological improvement associated with exercise in AUD (Colledge et al., 2018). Our findings lend enthusiasm and support for this continued evaluation.

Significant improvement in visuospatial immediate recall was only evident in the gameplay-control condition during Phase I. Although this condition was not designed to necessarily enhance executive function, it still required utilizing visuospatial skills to use and improve gameplay across sessions. It is not unusual for videogames which rely on the utilization of complex visuospatial skills to result in improvement in visuospatial memory (Basak et al., 2008), and our gameplay control condition likely had a similar effect. In fact, improvement in visuospatial recall during the gameplay control condition may have washed out any expected VR-EFT effects on visuospatial recall during Phase II. Improvement in visuospatial recall was not the target of VR-EFT, but nonetheless should be examined in future trials that compare these conditions in head-to-head challenge vs. a phased approach that may wash out VR-EFT specific findings.

Finally, we also observed significant improvement in cognitive flexibility for all participants during Phase I. Given the non-specific effect of improvement by condition in cognitive flexibility, it is likely that improvement in this domain was related to practice effects rather than true improvement associated with the condition (Lemay et al., 2004; Pietrzak et al., 2010; Lubrini et al., 2020; Sicard et al., 2020). Managing practice effects is a major issue for future cognitive training trials to contend with.

Our study had some major limitations. Namely, this was a relatively small pilot study that was designed primarily to evaluate the feasibility and usability of a VR-EFT plus exercise intervention and was not designed to definitively evaluate the effect of VR-EFT vs. other interventions on cognitive function or symptoms of AUD and TBI. As such, the associations observed in cognitive function and symptom improvement are considered preliminary and hypotheses generating only. Our small sample size also did not allow for the consideration of gender identity, sex, age, or race/ethnicity in the analyses or include a longitudinal analysis of cognition or TBI/AUD related symptoms, both important areas for future research. An additional limitation of this report is the reliance on self-report measures to assess TBI symptoms and alcohol use—although self-report at present remains the standard for alcohol use outcome measurement in clinical trials (Falk et al., 2010; Fertig et al., 2012; Litten et al., 2012). Of note, we did not include a measure of the quality of life related to TBI, a valuable and common assessment among TBI studies.

There is also a high likelihood that this population may have one or more of many common co-occurring conditions in addition to AUD and TBI, including PTSD, depression, anxiety, chronic pain, and problematic substance use (Corrigan and Cole, 2008; Stein et al., 2015; Kulas and Rosenheck, 2018; Miller, 2018), which can also increase the risk for alcohol-related concerns (Taylor et al., 2012; Wisco et al., 2014; Roberts et al., 2016; Miller, 2018). We did not evaluate symptoms associated with these common co-occurring conditions on intervention feasibility, usability, satisfaction, or other exploratory outcomes (e.g., cognitive function, alcohol use), as it fell outside the scope of this small pilot study. Understanding the high co-occurrence of PTSD and TBI among Veterans, future studies should examine the impact of comorbid PTSD on these outcomes. Finally, we did not require a specific fitness level or stratify randomization on fitness level at study entry. As seen in Table 1, the exercise-only group had much lower Leisure Activity Scores than the gameplay control condition. Although this likely did not impact the results of the current study, future studies examining the benefits of exercise combined with cognitive training should take great care in addressing activity levels at study entry.

Despite these limitations, the study was successful in establishing the feasibility, acceptability, and usability of conducting a VR-EFT and exercise study among Veterans with co-occurring TBI and AUD. VR-related adverse events were minimal, usability was at least acceptable, satisfaction was high, and adherence and retention were generally good among all conditions. We expect that retention would have been greatly improved had it not been for COVID-19 related withdrawals. All of this lends support for the successful completion of a larger VR-EFT trial examining the additional benefits that physical activity may have on cognitive training.

In sum, we established preliminary indication that exercise combined with VR-EFT is associated with improvement in executive function domains that were targeted in as little as 3 weeks and nine sessions of exposure. In addition, there was an indication that exercise-only was associated with improvement in executive function and reductions in alcohol use and craving. These findings can be used to inform the development of a larger definitive study, one that also includes a head-to-head challenge vs. a phased approach, and one that examines longitudinal far-transfer effects (i.e., 6–12-month rates of alcohol use and alcohol craving). We would also recommend that future studies of physically active VR-based cognitive training preemptively plan to assess pre-treatment cognitive function, baseline fitness level, and examine the effect of delivering personalized VR-EFT focusing on each participant’s unique needs. In sum, the results of this study indicate the need for a larger controlled investigation to more definitively assess the efficacy of physically active VR-EFT to enhance treatment outcomes among treatment-seeking individuals with co-occurring AUD and TBI.

## Data Availability Statement

Restrictions apply to the datasets: the datasets for this manuscript are not publicly available, because they were created as part of Veteran’s Administration approved research. Requests to access the datasets should be directed to the primary author DP, david.pennington2@va.gov.

## Ethics Statement

The studies involving human participants were reviewed and approved by University of California, San Francisco Institutional Review Board, San Francisco Veterans Affairs Healthcare System, VA Research and Development Committee. The patients/participants provided their written informed consent to participate in this study.

## Author Contributions

All authors have contributed to design and preparation of the manuscript. DP for conceptualization, funding acquisition, resources, supervision, writing (initial draft, review, and editing), clinical data management, and data analysis. JR for writing (initial draft, review, and editing) and clinical data management. MC, EW, and SB for clinical data management and completion of the RCT. All authors contributed to the article and approved the submitted version.

## Conflict of Interest

The authors declare that the research was conducted in the absence of any commercial or financial relationships that could be construed as a potential conflict of interest.

## Publisher’s Note

All claims expressed in this article are solely those of the authors and do not necessarily represent those of their affiliated organizations, or those of the publisher, the editors and the reviewers. Any product that may be evaluated in this article, or claim that may be made by its manufacturer, is not guaranteed or endorsed by the publisher.
